# Supplementary material for: Collaborative clinical reasoning: a scoping review
Source: PeerJ. 2024 Mar 6;12:e17042. doi: 10.7717/peerj.17042 (PMC10924455; doi:10.7717/peerj.17042)
Supplement: Supplemental Information 1 [file peerj-12-17042-s001.docx]

**Supplementary Information**

**Table S1 Search Strategy**

| **Database** | **PsychInfo OVID** |
| --- | --- |
| **Time Range** | 2011-2020 |
| **Search String** | 1 (cooperative or collaborative or shared or team or collective).m_titl.  2 reasoning.m_titl.  3 (problem and solving).m_titl.  4 (decision and making).m_titl.  5 (clinical or medicine or medical).mp. [mp=title, abstract, heading word, table of contents, key concepts, original title, tests & measures, mesh]  6 limit 5 to abstracts  7 2 or 3 or 4  8 1 and 6 and 7  9 limit 8 to yr="2011 - 2020"  10 (((reasoning or (problem and solving) or (decision and making)) and (cooperative or collaborative or shared or team or collective)).ti. and (clinical or medicine or medical).ab.) not (shared and decision and making).ti.  11 limit 10 to (english and yr="2011 - 2020") |

**Table S2 Categorisation of 24 collaborative clinical reasoning articles into journal genres**

| **Genres**    **Journals** | **Oncology** | **Nursing** | **Medical Education** | **ERGONOMICS**  **or**  **Medical Informatics** | **Psychology &Philosophy** | **Medicine in general** |
| --- | --- | --- | --- | --- | --- | --- |
| Annals of Surgical Oncology | 2 |  |  |  |  |  |
| Behaviour & Information Technology |  |  |  | 1 |  |  |
| BMC Cancer | 1 |  |  |  |  |  |
| BMC Medical Informatics & Decision Making |  |  |  | 1 |  |  |
| Cancer Medicine | 1 |  |  |  |  |  |
| Clinical Infectious Diseases |  |  |  |  |  | 1 |
| Communication and Medicine |  |  |  |  |  | 1 |
| Diagnosis |  |  |  |  |  | 1 |
| European Journal of Cancer Care | 1 |  |  |  |  |  |
| European Journal of Oncology Nursing | 1 |  |  |  |  |  |
| International Journal of Surgery |  |  |  |  |  | 1 |
| Journal of Advanced Nursing |  | 1 |  |  |  |  |
| Journal of Clinical Nursing |  | 1 |  |  |  |  |
| Journal of Continuing  Education in the  Health Professions |  |  | 1 |  |  |  |
| Journal of Geriatric Oncology | 1 |  |  |  |  |  |
| Medical Teacher |  |  | 1 |  |  |  |
| Medicine |  |  |  |  |  | 1 |
| Mind Culture and Activity |  |  |  |  | 1 |  |
| Oncology Research and Treatment | 1 |  |  |  |  |  |
| Plos One |  |  |  |  |  | 1 |
| Postgraduate Medical Journal |  |  | 1 |  |  |  |
| Synthese |  |  |  |  | 1 |  |
| World Journal of Surgery |  |  |  |  |  | 1 |
| **Total** | **8** | **2** | **3** | **2** | **2** | **7** |
